# Ultra-processed food consumption in Barbados: evidence from a nationally representative, cross-sectional study

**DOI:** 10.1017/jns.2021.21

**Published:** 2021-04-22

**Authors:** Rachel M. Harris, Angela M. C. Rose, Suzanne Soares-Wynter, Nigel Unwin

**Affiliations:** 1 The George Alleyne Chronic Disease Research Centre, Caribbean Institute for Health Research, The University of the West Indies, Bridgetown, Barbados; 2 Faculty of Medical Sciences, The University of the West Indies, Bridgetown, Barbados; 3 Epidemiology Department, Epiconcept, Paris, France; 4 Tropical Metabolism Research Unit, Caribbean Institute for Health Research, The University of the West Indies, Kingston, Jamaica; 5 MRC Epidemiology Unit, University of Cambridge, Cambridge, UK; 6 European Centre for Environment and Human Health, University of Exeter Medical School, Truro, UK

**Keywords:** Barbados, Diet, Sugar-sweetened beverages, Ultra-processed food, CARICOM, Caribbean community and common market, CI, Confidence interval, CROSQ, CARICOM Regional Organisation for Standards and Quality, DRs, Dietary recalls, HotN, Health of the Nation survey, NCDs, Non-communicable diseases, PAHO, Pan American Health Organization, SSBs, Sugar-sweetened beverages, UPFs, ultra-processed foods, USDA, United States Department of Agriculture, WHO, World Health Organization

## Abstract

Our objective was to describe, for the first time in an English-speaking Caribbean country, the contribution of ultra-processed foods (UPFs) to nutrients linked to non-communicable disease. Using a cross-sectional study design, dietary data were collected from two non-consecutive 24-h dietary recalls. Recorded food items were then classified according to their degree of processing by the NOVA system. The present study took place in Barbados (2012–13). A representative population-based sample of 364 adult Barbadians (161 males and 203 females) aged 25–64 years participated in the study. UPFs represented 40⋅5 % (838 kcal/d; 95 % CI 791, 885) of mean energy intake. Sugar-sweetened beverages made the largest contribution to energy within the UPF category. Younger persons (25–44 years) consumed a significantly higher proportion of calories from UPF (NOVA group 4) compared with older persons (45–64 years). The mean energy shares of UPF ranged from 22⋅0 to 58⋅9 % for those in the lowest tertile to highest tertile. Within each tertile, the energy contribution was significantly higher in the younger age group (25–44 years) compared with the older (45–64 years). One-quarter of persons consume ≥50 % of their daily calories from UPF, this being significantly higher in younger persons. The ultra-processed diet fraction contained about six times the mean of free sugars and about 0⋅8 times the dietary fibre of the non-ultra-processed fraction (NOVA groups 1–3). Targeted interventions to decrease the consumption of UPF especially in younger persons is thus of high priority to improve the diet quality of Barbadians.

## Introduction

Globally, dietary patterns are undergoing change with traditional diets based on minimally processed foods^(1,2)^ being displaced by convenient, mass-produced, highly marketed, ultra-processed foods (UPFs)^(3)^. The increased global capacity to produce and trade foods has led to this steady increase in the availability and consumption of UPF worldwide^(2,4,5)^. UPFs are by design microbiologically safe^(6)^, highly palatable, convenient^(1)^, with additives and cosmetic agents^(4)^ often inducing overconsumption of these foods^(7)^. The NOVA (a name, not an acronym) system is considered to be the most widely used and comprehensive food classification system^(8)^ which classifies foods and drinks based on the nature, extent and purpose of industrial food processing into four groups: unprocessed or minimally processed foods, processed culinary ingredients, processed foods and UPF^(1,2)^.

The Pan American Health Organization^(5)^ and the United Nations Food and Agricultural Organization^(9)^ propose the use of the percentage of energy intake derived from UPF as an index of the overall nutritional quality of contemporaneous diets. In developed countries such as Canada, an estimated 45 % of total daily energy intake comes from UPF; being higher among men, younger adults and those with fewer years of formal education^(10)^. While for Mexico, a developing nation, 30 % of total daily energy is attributed to UPF consumption; being highest in younger age groups and persons of higher educational levels^(11)^.

Increased inclusion of UPF results in nutritionally unbalanced diets^(10,12,13)^, higher in fat, salt and sugar^(14)^ with lower dietary fibre and vitamin densities^(15,16)^. These dietary imbalances increase the prevalence of cardio-metabolic risk factors such as hypertension, dyslipidaemia^(17,18)^, obesity^(19–22)^ and a raised glycaemic response^(23)^. In Latin America, as sales per capita of UPF have risen, the rates of overweight and obesity have been steadily increasing^(5)^.

The Seguimiento Universidad de Navarra (SUN) prospective cohort study set in Spain (*n* 19 899; aged 20–91 years) concluded that a high consumption of UPF (>4 servings/d) was independently associated with a 62 % increase risk (hazard ratio 1⋅62; 95 % CI 1⋅13, 2⋅33) for all-cause mortality^(24)^. This finding is further supported by two cohort studies, the NutriNet-Sante in France^(25)^ and the US, National Health and Nutrition Examination Survey (NHANES)^(26)^ which also found an association between UPF consumption and all-cause mortality.

Despite this growing evidence, the consumption of UPF has never been studied in an English-speaking Caribbean population. Previous estimates of UPF consumption in Latin America are derived from sales data^(5)^ and not from the assessment of dietary intake. Small island developing states like Barbados face particular challenges related to a markedly increasing reliance on food imports over the past 30 years, associated loss of local agricultural capacity, increasing vulnerability to climate change and extreme weather events, and rising burden of non-communicable diseases (NCDs). For Barbadians, between 30 and 70 years, women have a one-in-eight, and men have a one-in-five, the probability of dying from an NCD^(27)^. With this disease burden in mind, we describe in this novel study the contribution of UPF to the total daily energy intake and examine the influence of age, sex and highest level of education on UPF consumption. We also compare nutrient levels with recommended international nutrient guidelines for the prevention of NCDs^(28)^. The findings presented in the present paper complement existing knowledge on nutrient adequacy for this population^(29,30)^. These data provide local evidence which can be used to inform future public health nutrition policy in Barbados.

## Methods

### Data source and collection

The Barbados National Salt Study population was a subsample of the Barbados Health of the Nation (HotN) survey, a cross-sectional survey conducted in 2011–13, which recruited a nationally representative sample of adults aged ≥25 years (*n* 1234). The multistage probability sampling, recruitment and data collection methods for the present study have been previously described^(29–31)^. Briefly, a sample of 441 adults (aged 25–64 years) was randomly selected, stratified by sex and age group (25–44 and 45–64 years) with the aim of recruiting at least 100 persons in each group. Socio-demographic variables (age, sex and highest level of education) had already been collected during the HotN survey. Education was stratified into two categories: less than tertiary and tertiary. Tertiary education was defined as post-secondary education including college, vocational and university training. Age was grouped into older (45–64 years) and younger (25–44 years) age groups. Pregnant and lactating women were excluded because of their unique nutritional requirements.

### Procedures

Each participant completed two interviewer-administered, 24-h dietary recalls (DRs) collected on non-consecutive days. Trained data collectors, during face-to-face interviews, used the United States Department of Agriculture (USDA) Multi-Pass Method to record all food and beverages consumed on two non-consecutive days that fell within the same week. We aimed to capture the participants’ diet on one-week day and one weekend day. Information on serving sizes and portions consumed in one sitting were estimated using three-dimensional Nasco food models (Nasco Company, 901 Jamesville Ave, Fort Atkinson, WI, USA), standard measuring cups and household utensils. Reported foods were coded and recorded portions converted into grams by a Registered Dietitian (author R. M. H.). These data were double entered into the nutrition software, Nutribase Pro (version 9, Cybersoft Inc., 2016 E. Muirwood Drive, Phoenix, AR, USA). The underlying databases for this program are the USDA and Canadian food composition tables. The Association of Official Agricultural Chemists (AOAC) method was used to estimate dietary fibre^(32)^. Standardised traditional Barbadian recipes^(33)^ were added to the underlying food composition database, making this software more culturally appropriate.

### Food classification according to processing

We utilised all available dietary intake data for each participant using means of both DR days. Recorded food items (*N* 8329) were classified by Registered Dietitians (authors R. M. H. and S. S. W.) and an epidemiologist (author A. M. C. R.) according to the NOVA system, a classification based on the nature, extent and purpose of industrial food processing^(6)^. The NOVA system classifies all foods into four groups (1–4): ‘unprocessed or minimally processed foods’, ‘processed culinary ingredients’, ‘processed foods’ and ‘ultra-processed foods’. Each of these four NOVA groups was further divided into subgroups^(6)^.

The first NOVA group, for unprocessed or minimally processed foods, contained thirteen subgroups including fresh, dried, ground, chilled, frozen, pasteurised or fermented staple foods. The second NOVA group, which included processed culinary ingredients contained four subgroups including salt, plant oils, butter, sugar, honey and other substances extracted from foods and used in kitchens to transform unprocessed or minimally processed foods into culinary preparations.

The third NOVA group defined the processed foods group. The six subgroups within this included foods such as canned or bottled vegetables, fruits and legumes, salted, cured or smoked meat, canned fish and cheeses. In the fourth NOVA group, comprising UPFs, sixteen subgroups were defined, which included industrialised packaged breads, sugar-sweetened beverages (SSBs), packaged prepared meals (instant noodles) breakfast cereals and other reconstituted meat products (hotdogs, nuggets and burgers). The ingredients of UPF which were classified in this group included substances not normally or never used in culinary preparations, such as modified starches, hydrogenated oils, hydrolysed protein and additives (e.g. emulsifiers, humectants, sequestrants, and firming, bulking, de-foaming, anti-caking and glazing agents). Further information detailing the NOVA food classification can be found in Supplementary Table S1 of Supplementary material^(34)^.

The traditional staple starchy foods in the Barbadian diet of green banana, breadfruit and plantain were classified within the subgroup of ‘starchy roots and tubers’ within NOVA group 1. We justified this subgrouping because these starchy fruits are eaten as staples in the Caribbean diet falling within the Caribbean food grouping of ‘starchy roots, fruits and tubers’^(35)^. The category of ‘legumes’ was expanded to include nuts, seeds and raw coconut (*n* 5). Again, we justified this categorisation as the Caribbean food grouping of ‘legumes’ includes nuts and seeds^(35)^. When uncertainty existed about a food or beverage item, we reached a consensus based on the percentage of homemade and artisanal foods *v.* industrial brands of processed and UPF reported by the participants. A few freshly prepared traditional Barbadian composite dishes (*n* 21), based mainly on unprocessed or minimally processed foods, were not disaggregated but classified as NOVA group 1 and then categorised by subgroup according to their main ingredient.

### Data analysis

We used the mean of the 2 d of dietary data for each person. The distributions of the variables of interest were approximately normal, and we, therefore, present mean values with their corresponding 95 % confidence intervals (CI). First, we estimated for each participant the total energy intake by each of the four NOVA food groups and subgroups. The mean daily energy intakes by NOVA food group and subgroups for the sample were generated. Secondly, the energy intakes for each participant were then standardised to a 2000 kcal/d intake, and the distribution according to the NOVA grouping detailed.

As expected, there was a range of total dietary energy intakes for both men and women. Standardisation enables the relative contribution of different food groups to be compared across population subgroups while controlling for confounding by the total energy intake^(36)^. Within each NOVA group for both the total and standardised energy intake estimates a comparison by age group (older *v.* younger), sex (male *v.* female) and education (tertiary *v.* non-tertiary) using the Student's *t* test was performed.

We then evaluated the prevalence of inadequate intake across two diet fractions, namely UPF (NOVA group 4) and non-UPF fractions (NOVA groups 1–3), using the international dietary nutrient recommendations specified by the World Health Organization (WHO)^(28)^. For overall diet and across these two diet fractions, the mean macronutrient content (expressed as the percent of total energy), sodium, potassium (expressed as mg/1000 kcal) and dietary fibre (expressed as g/1000 kcal) intakes were estimated. The energy density from the solid fraction of the diet was calculated by dividing the sum of calories from solid food (excluding all beverages) by the weight in grams of these foods. The recommendation for energy density, as proposed by the World Cancer Research Fund, was used for comparison^(37)^.

Lastly, we stratified the sample into tertiles according to the percentage of energy derived from UPF in an individual's diet, with the lowest consumers in the first tertile and the highest in the third. The contribution of each NOVA food (sub)group across tertiles of the dietary energy contribution of UPF (i.e. the energy shares of UPF) was described. We then examined differences within each tertile by age, sex and educational level using the Student's *t* test.

Statistical significance is reported as *P* < 0⋅05. All statistical analyses were carried out using STATA v15 (StataCorp. LP, College Station, TX, USA) taking into account the cluster survey design, non-response and to match the age and sex distribution of the Barbadian population according to the 2010 Barbados and Population Housing Census^(38)^.

## Results

For the present study, 441 Barbadian adults were selected from the participants in the HotN, of which 364 (83 % response rate) consented to take part. The final sample comprised 203 (55⋅8 %) women and 161 (44⋅2 %) men. The data for five participants were excluded due to missing variables. The final sample size for all analyses was 359 (200 women and 159 men) participants (Table 1).

**Table 1.** Demographic characteristics of the Barbados National Salt Study (BNSS) sample by age group, sex, and educational level (2012–13)

	Women (*n* 200)		Men (*n* 159)		Overall (*N* 359)	
	Number	%^a^	Number	%^a^	Number	%^a^
Age group (Years)						
25–44	89	51⋅9	77	54⋅0	166	52⋅9
45–64	111	48⋅1	82	46⋅0	193	47⋅1
Education					*N* 358	
<Tertiary	151	76⋅8	118	77⋅0	226	76⋅9
Tertiary	49	23⋅2	40^b^	23⋅0	138	23⋅1

Tertiary refers to all post-secondary school education, including technical and vocational training as well as university course.

a Percentages are weighted to compensate for unequal probabilities of selection (selecting one individual from household) and for non-response.

b Information on educational level was missing for one individual (*n* 358).

Table 2 details the mean estimated daily energy intake by the NOVA group. As shown in Table 2, the greatest proportions came from unprocessed or minimally processed food (47 %; 969 kcal; 95 % CI 916, 1021) and UPFs (41 %; 838 kcal; 95 % CI 791, 885), with the other two NOVA groups together compromising 12 %. In the Barbadian diet, about two-thirds of the energy within NOVA group 1 (unprocessed or minimally processed foods) came from four subgroups: cereals, poultry, pasta and starchy roots and tubers. About one-third of energy in unprocessed or minimally processed foods came from foods of animal origin. Table sugar in the NOVA group 2 of processed culinary ingredients contributed the most towards total energy accounting for three-quarters of the total share within this NOVA group. Within processed foods (NOVA group 3), the main contributors were other processed foods, salted/smoked/canned meats/fish, beer and wine, together contributing about three-quarters of the total energy in this group. The top three contributors in the UPF group (NOVA group 4) were SSBs, industrial packaged bread, and pastries, buns and cakes, which collectively made up over half of the energy for this group. The subgroup of SSBs (soft drinks, fruit drinks and juices) accounted for 21 % of energy within the UPF group (Table 2).

**Table 2.** Distribution of mean daily energy intake by NOVA food groups in adult Barbadians (2012–13)

NOVA food groups and subgroups	Mean, kcal/d (95 % CI)	% of total energy intake
Unprocessed or minimally processed foods	968⋅7 (916⋅3, 1021⋅1)	46⋅8
Cereals	209⋅2 (188⋅1, 230⋅3)	10⋅1
Poultry	167⋅7 (114⋅7, 190⋅7)	8⋅1
Pasta	125⋅0 (100⋅3, 149⋅8)	6⋅0
Starchy roots and tubers	116⋅3 (97⋅4, 135⋅2)	5⋅6
Red meat	90⋅2 (7⋅5, 110⋅0)	4⋅4
Fruits	72⋅9 (60⋅3, 85⋅6)	3⋅5
Fish	56⋅5 (43⋅5, 69⋅4)	2⋅7
Vegetables	36⋅2 (30⋅5, 41⋅8)	1⋅7
Legumes	33⋅9 (23⋅2, 44⋅6)	1⋅6
Eggs	23⋅4 (18⋅5, 28⋅4)	1⋅1
Fresh fruit juice	17⋅3 (11⋅0, 23⋅7)	0⋅8
Other un- or minimally processed foods^a^	13⋅0 (9⋅8, 16⋅2)	0⋅6
Milk and plain yoghurt	7⋅3 (4⋅3, 10⋅3)	0⋅4
Processed culinary ingredients	24⋅1 (19⋅9, 28⋅2)	1⋅2
Table sugar	18⋅6 (15⋅4, 21⋅9)	0⋅9
Butter	4⋅2 (1⋅6, 6⋅9)	0⋅2
Plant oil	0⋅6 (0⋅1, 1⋅2)	0⋅0
Other processed culinary ingredients^b^	0⋅6 (0⋅1, 1⋅1)	0⋅0
Processed foods	240⋅3 (211⋅8, 268⋅7)	11⋅6
Other processed foods^c^	86⋅8 (73⋅1, 100⋅6)	4⋅2
Ham and salted/smoked/canned meats/fish	51⋅4 (41⋅5, 61⋅3)	2⋅5
Beer and wine	43⋅4 (23⋅2, 63⋅6)	2⋅1
Cheese	33⋅5 (26⋅3, 40⋅6)	1⋅6
Processed breads	22⋅3 (14⋅2, 30⋅3)	1⋅1
Canned or bottled vegetables/fruit/legumes	2⋅9 (0⋅2, 5⋅7)	0⋅1
Ultra-processed foods	838⋅1 (791⋅0, 885⋅3)	40⋅5
Soft drinks, fruit drinks/juices	173⋅3 (159⋅5, 187⋅2)	8⋅4
Industrialised packaged breads	142⋅6 (127⋅9, 157⋅3)	6⋅9
Pastries, buns and cakes	125⋅3 (104⋅1, 146⋅5)	6⋅0
Other ultra-processed foods^d^	86⋅3 (73⋅7, 98⋅9)	4⋅2
Industrial chips (French fries)	61⋅9 (47⋅9, 75⋅8)	3⋅0
Sausage and other reconstituted meat products	57⋅9 (47⋅7, 68⋅0)	2⋅8
Milk-based drinks	32⋅8 (25⋅9, 39⋅7)	1⋅6
Confectionary	33⋅6 (24⋅8, 42⋅5)	1⋅6
Breakfast cereals	31⋅3 (21⋅7, 40⋅9)	1⋅5
Biscuits	25⋅9 (18⋅7, 33⋅1)	1⋅3
Industrial pizza	22⋅1 (14⋅0, 30⋅2)	1⋅1
Packaged snacks	14⋅1 (8⋅8, 19⋅3)	0⋅7
Industrial desserts	11⋅1 (5⋅8, 16⋅5)	0⋅5
Packaged pre-prepared meals	9⋅5 (4⋅1, 15⋅0)	0⋅5
Margarine, other spreads	6⋅5 (4⋅2, 8⋅9)	0⋅3
Sauces, dressings, gravies	3⋅9 (2⋅3, 5⋅5)	0⋅2
Total	2071⋅2 (1939⋅0, 2203⋅4)	100⋅0

a Including meat from other animals, teas, coffees and dried spices.

b Including honey, gelatine powder and vinegar.

c Including salted or sugared nuts, seeds and dried fruit.

d Including ultra-processed cheese.

Table 3 summarises the result from the Student's *t* test comparisons within each NOVA group. Men consumed a statistically significantly higher proportion of calories from NOVA group 3 compared with women. Age impacts on the proportion of calories being significantly higher in the older age group (45–64 years) for NOVA groups 1 and 2. Younger persons (25–44 years) consume a significantly higher proportion of calories of UPF (NOVA group 4) compared with older persons (45–64 years). UPF consumption was higher for persons not having tertiary education; however, the influence of educational level on the proportion of calories was not statistically significant in any of the NOVA groups.

**Table 3.** Distribution of standardised energy intake to 2000 kcal/d according to the NOVA food groups, by age, sex, and educational level groups in adult (25–64 years) Barbadians (2012–13)

NOVA group	Mean energy intake, kcal/d (95 % CI)			
	1	2	3	4
Sex				
Men	925⋅0 (870⋅4, 979⋅6)	22⋅8 (15⋅7, 30⋅0)	**249**⋅**8** (**214**⋅**4, 285**⋅2)*	802⋅4 (750⋅8, 854⋅1)
Women	956⋅3 (906⋅4, 1006⋅2)	26⋅9 (21⋅8, 32⋅0)	**205**⋅**5** (**180**⋅**3, 230**⋅**8)**	811⋅3 (763⋅4, 859⋅2)
Age (Years)				
25–44	**877**⋅**0** (**825**⋅**8, 928**⋅**2)**	**18**⋅**9 (13**⋅**8, 24**⋅**0)**	215⋅1 (186⋅3, 243⋅8)	**889**⋅**1** (**835**⋅**8, 942**⋅3)*
45–64	**998**⋅**7** (**947**⋅**5, 1049**⋅9)*	**30**⋅**4** (**23**⋅**9, 37**⋅0)*	233⋅8 (203⋅2, 264⋅4)	**737**⋅**1** (**692**⋅**8, 781**⋅**3)**
Educational level				
<Tertiary	924⋅7 (882⋅4, 967⋅0)	26⋅0 (20⋅9, 31⋅1)	230⋅9 (206⋅4, 255⋅3)	818⋅5 (778⋅4, 858⋅5)
Tertiary	999⋅8 (925⋅4, 1074⋅3)	22⋅1 (14⋅4, 29⋅7)	209⋅8 (167⋅3, 252⋅3)	768⋅3 (696⋅0, 840⋅7)

* *P* < 0⋅05. Statistically significant mean values in bold type.

Approximately one-in-five (18⋅7 %) Barbadians in the present study obtained less than 25 % of their daily calories from UPF, primarily in older adults (45–64 years; *P* = 0⋅006). One-in-four persons (27⋅9 %) consume more than 50 % of their daily calories from UPF, this occurs primarily in younger adults (25–44 years) than older adults (45–64 years; *P* = 0⋅000) (data not shown).

In Table 4, the nutrient profiles of the overall diet, the non-UPF (NOVA groups 1–3) and UPF diet fractions (NOVA group 4) of the Barbadian diet are compared with the WHO dietary intake recommendations for key nutrients. The UPF diet fraction contained about six times the mean of free sugars and about 0⋅8 times the dietary fibre of the non-UPF fraction. The percentage of total energy from fat, saturated fat and trans-fats was within the recommendations. The percentage energy from protein was above the recommended range for both diet fractions, being highest in the non-UPF fraction (21⋅7 %). The mean energy density was highest in the UPF diet fraction (2⋅9 kcal/g) compared with the non-UPF fraction (1⋅3 kcal/g). Sodium intake was also higher than recommended, being highest in the non-UPF than the UPF fraction. Inadequate intakes for dietary fibre and potassium were observed across both diet fractions.

**Table 4.** Average nutrient content for the overall diet and UPF *v.* other diet fractions combined, and compared with the WHO dietary intake recommendations for key nutrients, in adult Barbadians (2012–13)

Nutrient indicators	Overall diet	Non-ultra-processed food diet fraction^a^	Ultra-processed food diet fraction	Recommended values for the indicators
	Mean (95 % CI)	Mean (95 % CI)	Mean (95 % CI)	
Free sugars (% of total energy)	15⋅6 (14⋅8, 16⋅4)	5⋅2 (4⋅6, 5⋅8)	32⋅0 (30⋅2, 33⋅8)	<10^b^
Total fats (% of total energy)	28⋅4 (27⋅6, 29⋅2)	30⋅6 (29⋅6, 31⋅6)	24⋅8 (23⋅6, 26⋅0)	15–30^b^
Saturated fats (% of total energy)	8⋅0 (7⋅6, 8⋅4)	9⋅0 (8⋅6, 9⋅4)	6⋅3 (5⋅9, 6⋅7)	<10^b^
Trans-fats (% of total energy)	0⋅1 (0⋅1, 0⋅1)	0⋅0 (0⋅0, 0⋅1)	0⋅2 (0⋅1, 0⋅2)	<1^b^
Protein (% of total energy)	16⋅7 (16⋅1, 17⋅3)	21⋅7 (20⋅9, 22⋅5)	9⋅6 (9⋅0, 10⋅2)	10–15^b^
Energy density (kcal/g)	1⋅6 (1⋅5, 1⋅7)	1⋅3 (1⋅2, 1⋅4)	2⋅9 (2⋅8, 3⋅0)	1⋅25–1⋅45^c^
Dietary fibre (g/1000 kcal)	9⋅7 (9⋅3, 10⋅0)	10⋅4 (9⋅8, 11⋅0)	8⋅6 (8⋅0, 9⋅2)	>12⋅5^d^
Sodium (mg/1000 kcal)	1427⋅7 (1381⋅2, 1474⋅2)	1522⋅1 (1459⋅6, 1584⋅6)	1293⋅2 (1238⋅9, 1347⋅5)	<1000 mg^e^
Potassium (mg/1000 kcal)	1283⋅3 (1236⋅7, 1329⋅9)	1666⋅7 (1592⋅2, 1741⋅2)	722⋅1 (684⋅3, 759⋅9)	>1755 mg^e^

a Includes the NOVA unprocessed or minimally processed foods, processed culinary ingredients and processed foods (NOVA groups 1–3).

b World Health Organization (2013) Diet, nutrition, and the prevention of chronic disease. Geneva: World Health Organization.

c World Cancer Research Foundation (2009) Energy density: finding the balance for cancer prevention. London: World Cancer Research Foundation.

d Recommended value based on a 2000 kcal diet.

e World Health Organization (2013) World Health Organization issues new guidance on dietary salt and potassium. Geneva: WHO. Recommended value based on a 2000 kcal diet.

Table 5 summarises the tertiles of the energy share of UPFs. The mean energy shares of UPF ranged from 22⋅0 % (95 % CI 14⋅6, 29⋅4) of total daily intake for those in the lowest tertile to 58⋅9 % (95 % CI 50⋅0, 67⋅7) in the highest tertile. This marked difference between tertiles 1 and 3 in the NOVA 4 group results from the higher contribution made to energy by the subgroups of SSBs, pastries, buns and cakes, and industrialised packaged breads within tertile 3. In tertile 3 for NOVA group 4, the energy contribution is above 50 % (58⋅9 %; 95 % CI 50⋅0, 67⋅7). The energy contribution of all subgroups belonging to UPF (NOVA 4) increased from the first to the third tertile. The opposite was observed among all other subcategories in NOVA groups 1–3, except for ‘other foods’ and cheese in the processed food group (NOVA 3) and fresh fruit juice milk and plain yoghurt in the unprocessed or minimally processed foods (NOVA 1). From the lowest to the highest UPF tertiles, the energy share increased by a factor of eight for packaged pre-prepared meals, by a factor of six for pizza, approximately five for biscuits and industrial desserts, and approximately four for pastries, buns and cakes, confectionary and industrial chips (French fries). Within each tertile, the energy contribution was significantly higher in the younger age group (25–44 years) compared with the older (45–64 years) (data not shown). Sex and educational level were not significant.

**Table 5.** Tertiles of the energy share of ultra-processed foods in adult (25–64 years) Barbadians (2012–13)

NOVA food groups	Tertiles of the dietary contribution of ultra-processed food (% of the dietary energy)		
	T1	T2	T3
	(*n* 120)	(*n* 120)	(*n* 119)
Unprocessed or minimally processed foods	62⋅4 (95 % CI 53⋅8, 71⋅1)	47⋅6 (95 % CI 38⋅6, 56⋅5)	31⋅1 (95 % CI 22⋅8, 39⋅4)
Cereals	12⋅5	11⋅3	6⋅8
Poultry	10⋅6	8⋅4	5⋅4
Starchy roots and tubers	8⋅4	5⋅0	3⋅6
Pasta	8⋅5	6⋅4	3⋅5
Fruits	5⋅1	2⋅7	2⋅8
Red meat	6⋅6	4⋅1	2⋅6
Fish	3⋅6	2⋅6	2⋅0
Vegetables	2⋅1	1⋅8	1⋅3
Fresh fruit juice	0⋅7	0⋅9	1⋅0
Eggs	1⋅5	1⋅2	0⋅8
Legumes	2⋅1	2⋅3	0⋅6
Other^a^	0⋅7	0⋅7	0⋅5
Milk and plain yoghurt	0⋅3	0⋅3	0⋅4
Processed culinary ingredients	1⋅3 (95 % CI 0⋅7, 3⋅3)	1⋅3 (95 % CI 0⋅7, 3⋅4)	0⋅9 (95 % CI 0⋅8, 2⋅6)
Table sugar	0⋅9	1⋅0	0⋅7
Butter	0⋅3	0⋅2	0⋅2
Plant oil	0⋅0	0⋅1	0⋅0
Other^b^	0⋅1	0⋅0	0⋅0
Processed foods	14⋅3 (95 % CI 8⋅1, 20⋅6)	11⋅4 (95 % CI 5⋅8, 17⋅1)	9⋅1 (95 % CI 4⋅0, 14⋅3)
Other^c^	3⋅7	4⋅8	3⋅9
Cheese	1⋅3	1⋅9	1⋅8
Ham and salted/smoked/canned meats/fish	3⋅1	3⋅0	1⋅7
Beer and wine	4⋅5	0⋅8	0⋅9
Processed breads	1⋅3	1⋅0	0⋅7
Canned or bottled vegetables/fruit/legumes	0⋅5	0⋅1	0⋅1
Ultra-processed foods	22⋅0 (95 % CI 14⋅6, 29⋅4)	39⋅7 (95 % CI 30⋅9, 48⋅4)	58⋅9 (95 % CI 50⋅0, 67⋅7)
Soft drinks, fruit drinks/juices	5⋅8	8⋅4	10⋅6
Pastries, buns and cakes	2⋅4	5⋅2	10⋅4
Industrialised packaged breads	4⋅2	7⋅2	9⋅2
Industrial chips (French fries)	1⋅3	2⋅5	5⋅2
Other^d^	2⋅9	4⋅7	5⋅0
Sausage and other reconstituted meat products	1⋅4	2⋅7	4⋅3
Confectionary	0⋅6	1⋅7	2⋅6
Breakfast cereals	1⋅0	0⋅9	2⋅6
Milk-based drinks	0⋅8	1⋅8	2⋅1
Industrial pizza	0⋅3	0⋅9	2⋅0
Biscuits	0⋅4	1⋅4	1⋅9
Industrial desserts	0⋅2	0⋅4	0⋅9
Packaged snacks	0⋅4	0⋅8	0⋅8
Packaged pre-prepared meals	0⋅1	0⋅5	0⋅8
Margarine and other spreads	0⋅2	0⋅4	0⋅4
Sauces, dressings and gravies	0⋅2	0⋅1	0⋅3

a Including meat from other animals, teas, coffees and dried spices.

b Including honey, gelatine powder and vinegar.

c Including salted or sugared nuts, seeds and dried fruit.

d Including ultra-processed cheese.

## Discussion

In this analysis of nationally representative data, we found that almost 40⋅5 % (838⋅1 kcal; 95 % CI 791⋅0, 885⋅3) of dietary energy came from UPF, with three subgroups (SSBs, industrial packaged breads, and pastries, buns and cakes) making the greatest contribution. This energy share of UPF to the total energy in the Barbadian diet is similar to levels found in high-income developed countries, such as the United States (58 %)^(13)^ and Canada (48 %)^(39)^. Similar estimates have been notably lower in low- and middle-income countries such as Brazil (20 %)^(22)^ and Mexico (30 %)^(11)^, where traditional diets based on freshly prepared meals and minimally processed foods still predominate.

Habitual consumption of diets high in free sugars and processed carbohydrates tend to be energy-dense, low in satiety-promoting fibre, cheap, widely available and often heavily marketed, predisposing individuals to excess weight gain irrespective of the macronutrient profile^(40)^. Our study further confirms previous findings in Barbados, where the overconsumption of SSBs was described^(30)^. These obesogenic beverages have been shown to delay the trigger of the internal satiety signal, resulting in excessive caloric ingestion^(41)^. This reliance on SSBs could potentially drive an increase in the already alarmingly high levels of obesity (33⋅8 %; 95 % CI 18⋅9, 30⋅0)^(31)^ and cardiovascular disease in Barbadians^(42)^ especially in younger age groups.

The low levels of dietary fibre in the Barbadian diet can lead to the increased prevalence of obesity, diabetes, cardiovascular diseases^(43,44)^, and cancers of the colon, rectum and breast^(28)^. Health promotional efforts targeting an increase in the overall intake of dietary fibre through the increased consumption of fruits, vegetables, legumes and wholegrains are recommended especially for younger persons. The average protein content (16⋅7 % of total calories) exceeded the recommendation (10–15 %). The Barbadian diet is characterised by the regular consumption of protein derived mainly from meat, fish, cheese, eggs and legumes^(30,45)^. The adverse effects on health resulting from high protein intake remain unclear; however, high protein intakes have been linked to kidney function damage and conversely the increased satiety of the diet, thus reducing excessive calorie consumption^(46)^.

The higher sodium density in the non-UPF fraction can be partially explained by the fact that the traditional Barbadian cuisine is highly seasoned. Salt and other seasonings containing sodium (chopped seasoning, soya sauce, stock cubes and other condiments) are added during food preparation and cooking^(29)^. Educating the public on these hidden sources of salt and daily sodium intake recommendations is merited. Reformulation of food products by food manufacturers to reduce the sodium content is also recommended. The low intake of fruits and vegetables in Barbadians may partially explain the finding of inadequate potassium intake, found using the gold standard approach of 24- h urine collection and reported by the authors previously^(29)^. This is of concern as insufficient dietary potassium and excessive sodium increases the risk of high blood pressure^(47)^ the main risk factor for cardiovascular diseases.

Our study's strengths include the use of the most up-to-date, individual dietary survey data taken from a nationally representative sample of Barbadian adults. We applied weighting procedures to adjust for differences between the sample and population distributions, increasing the generalisability of our findings to the Barbadian population and other Caribbean nations with similar demographics and diet. All data were collected following internationally recognised, standardised procedures.

We do recognise, however, that although dietary data obtained by 24-h DRs are useful to estimate group means, they are subject to within-person variation and observational error, defined as the difference between the measured diet and its true value^(48)^. Under-reporting of unhealthy foods may have led to an underestimation of the dietary contribution of UPF and the overall intake of some nutrients such as sodium, fat and free sugars. Our dataset did not contain a detailed ingredients list for composite foods, as our initial goal was to identify foods which could be targeted in a future food-based nutrition intervention. For this reason, recipes were not disaggregated. We, therefore, classified composite foods into NOVA food groups dependent on their main ingredients. We recognise this as a limitation in our analyses and study findings. This assumption could have introduced systematic error and measurement error, based on the misclassification of individual recipe components. Recorded food items may also have been misclassified due to inconsistencies of information indicative of food processing in the datasets. By examining diet fractions, we were able to overcome some of these limitations.

Our local food environment relies greatly on imported products^(49)^, with the pervasive marketing of processed foods driving their consumption. The high consumption of foods away from home and SSBs for this population has been previously described^(28,29)^. By targeting key domains of the food system: food composition, labelling, marketing, provision, retail, prices, and trade^(50)^ interventions for healthier food environments can be achieved. Using a modelling approach in the UK, a reduction in the consumption of UPF has been predicted to prevent or postpone approximately 10 % of cardiovascular deaths^(51)^. Urgent measures need to be taken to reverse this current situation in Barbados, especially in younger ages. Mandatory front-of-package labelling was introduced in Chile in 2016 after analysing food consumption, for all food products that are energy-dense or are high in sugar, saturated fat or sodium^(15)^. In November 2014, the Brazilian government issued national dietary guidelines that specify avoidance of ultra-processed products^(52)^.

To date, the Caribbean region activities are presently underway to include a Caribbean-wide mandatory front-of-package food labelling. This initiative is being spearheaded by the CARICOM Regional Organisation for Standards and Quality (CROSQ). Also in September 2015, Barbados introduced a 10 % excise tax on SSBs as an initial step towards reducing the overall population consumption^(53)^. The evaluation of the effectiveness of this fiscal policy has found that weekly sales of SSBs have been reduced by 4⋅3 %^(54)^. Continued public health nutrition policies and actions are required to reduce and reverse the displacement of unprocessed or minimally processed foods and meals, by UPF, beverages and snacks.

## Conclusions

To the best of our knowledge, this is the first study in the English-speaking Caribbean to examine the consumption of UPFs. The Barbadian diet is characterised by a pervasive and ubiquitous reliance on UPFs for both sexes and across all age groups. The higher consumption of UPF in younger Barbadians (25–44 years) may result in a cohort effect where they may maintain their high intake of UPF consumption as they get older. These findings contribute to the scarce existing literature on UPF consumption from a developing country setting. Future investigations into the cross-sectional relationships between UPF intake and anthropometric, and metabolic variables in Barbadians are warranted. From a public health perspective, our findings reinforce the urgent need for public health nutrition initiatives, education and policy engaging all sectors of the Barbadian population to encourage the consumption of minimally processed fresh foods for optimal health.

## Supplementary material

For supplementary material accompanying this paper visit http://dx.doi.org/10.1017/jns.2021.21. click here to view supplementary material
